# Peripheral versus central cannulation of VA-ECMO for primary graft dysfunction after heart transplantation: A systematic review and meta-analysis

**DOI:** 10.1016/j.jhlto.2024.100174

**Published:** 2024-11-08

**Authors:** Eduard Ródenas-Alesina, Aleix Olivella, Ani Orchanian-Cheff, Farid Foroutan, Yasbanoo Moayedi, Vivek Rao, Filio Billia, Heather J. Ross, Ana Carolina Alba, Natasha Aleksova

**Affiliations:** a Cardiology Department, Hospital Universitari Vall d′Hebron, Vall d′Hebron Institut de Recerca, Barcelona, Spain; b Centro de Investigación Biomédica en Red de Enfermedades Cardiovasculares (CIBERCV), Instituto de Salud Carlos III, Madrid, Spain; c Library and Information Services, University Health Network, Toronto, Ontario, Canada; d Peter Munk Cardiac Centre, Toronto General Hospital, University Health Network, Toronto, Ontario, Canada; e Women’s College Hospital, Toronto, Ontario, Canada; f Departament de Medicina, Universitat Autònoma de Barcelona, Barcelona, Spain

**Keywords:** VA-ECMO, primary graft dysfunction, heart transplantation, mechanical circulatory support, meta-analysis

## Abstract

**Background:**

Severe primary graft dysfunction (PGD) after heart transplantation (HT) is a major cause of death and requires veno-arterial extracorporeal membrane oxygenation (VA-ECMO).

**Methods:**

We conducted a systematic review and meta-analysis including studies of adult HT recipients who required VA-ECMO for PGD to determine whether a peripheral or central configuration was associated with higher mortality. The primary endpoints were short-term and one-year mortality. Secondary endpoints were VA-ECMO-related complications.

**Results:**

Overall, we included 16 studies comprising 874 patients from 33 centers. Using a random-effects model, peripheral cannulation was associated with a nonsignificant reduction in short-term mortality (odds ratios [OR] = 0.73, 95% confidence interval [CI] = 0.41-1.28, I2 = 55.8%) and a significant reduction in 1-year mortality (OR = 0.60, 95%CI = 0.37-0.97, I2 = 35.9%). Peripheral cannulation decreased the risk of bleeding but increased the risk of limb ischemia and infection, with similar rates of stroke and need for renal replacement therapy. Overall, certainty of evidence was low.

**Conclusions:**

With low certainty evidence, peripheral VA-ECMO cannulation may reduce short-term and 1-year mortality with lower bleeding rates but higher limb-related complications, supporting peripheral configuration in HT recipients with severe PGD.

## Background

Severe primary graft dysfunction (PGD) affects 7.8% of heart transplant (HT) recipients and portends a 1-year mortality close to 25%.^1^ In non-HT postcardiotomy shock, peripheral veno-arterial extracorporeal membrane oxygenation (VA-ECMO) cannulation has demonstrated higher survival than central cannulation,^2^, ^3^ but there still exists significant between-center variability in VA-ECMO configuration for PGD. Since the available evidence originates from small studies^4^ and a large multicentric study was recently published and not included in existent meta-analyses,^5^ we conducted an update of a previous systematic review and meta-analysis to determine complication rates according to the cannulation strategy (peripheral vs central VA-ECMO) in PGD.

## Methods

A research librarian (A.O.C.) executed the same comprehensive search strategy as previously reported^4^ until July 7, 2023. We included studies published after January 1, 2009, of adult (≥18 years old) HT recipients with PGD receiving VA-ECMO that reported data for any prespecified endpoint based on the site of cannulation. Studies with <5 patients or multiorgan recipients were excluded.

Title, abstract, and full-text screening were completed in duplicate (E.R.A. and A.O.S.), and in case of disagreement a third reviewer (N.A.) intervened. If there was overlapping data, only the most recent cohort was included. Individual patient data (IPD) was used when available.^4^

Outcomes of interest were short-term mortality (in-hospital mortality or 30-day mortality) and 1-year mortality. VA-ECMO-related complications were stroke (hemorrhagic or ischemic), bleeding, or infection while supported on VA-ECMO, renal replacement therapy (RRT), and limb ischemia.

Pooled effect sizes and odds ratios (OR) were calculated using random-effects restricted maximum likelihood models with the Freeman-Tukey double arcsine transformation (Stata 18.0, StataCorp). We conducted subgroup analyses according to study type (single-center vs multicenter), use of IPD, and International Society for Heart and Lung Transplantation (ISHLT) definition for PGD.^6^ The risk of bias was assessed using the ROBINS-I tool,^7^ and GRADE framework was used to assess the certainty of the evidence.^8^ Publication bias was assessed with funnel plots, and small studies effect with Egger’s test.

## Results

After removal of 117 duplicates from the updated search, 2,086 studies underwent title and abstract screening, and 102 were screened as full texts. We included 16 nonrandomized studies comprising 874 patients from 33 centers with recruitment timeframes ranging from 2000-2014 to 2008-2020. IPD was available for 10 studies.^4^ Fifteen studies were retrospective, 15 were published as full text, and 2 studies were multicentered. The ISHLT definition for PGD was used in 8 studies. The risk of bias was high for most studies for mortality (Table S1) because of inadequate control of confounding. However, most articles included consecutive patients with little missing data. There was no significant publication bias (Figure S1) nor small studies effect on any outcome.

**Figure 1:**
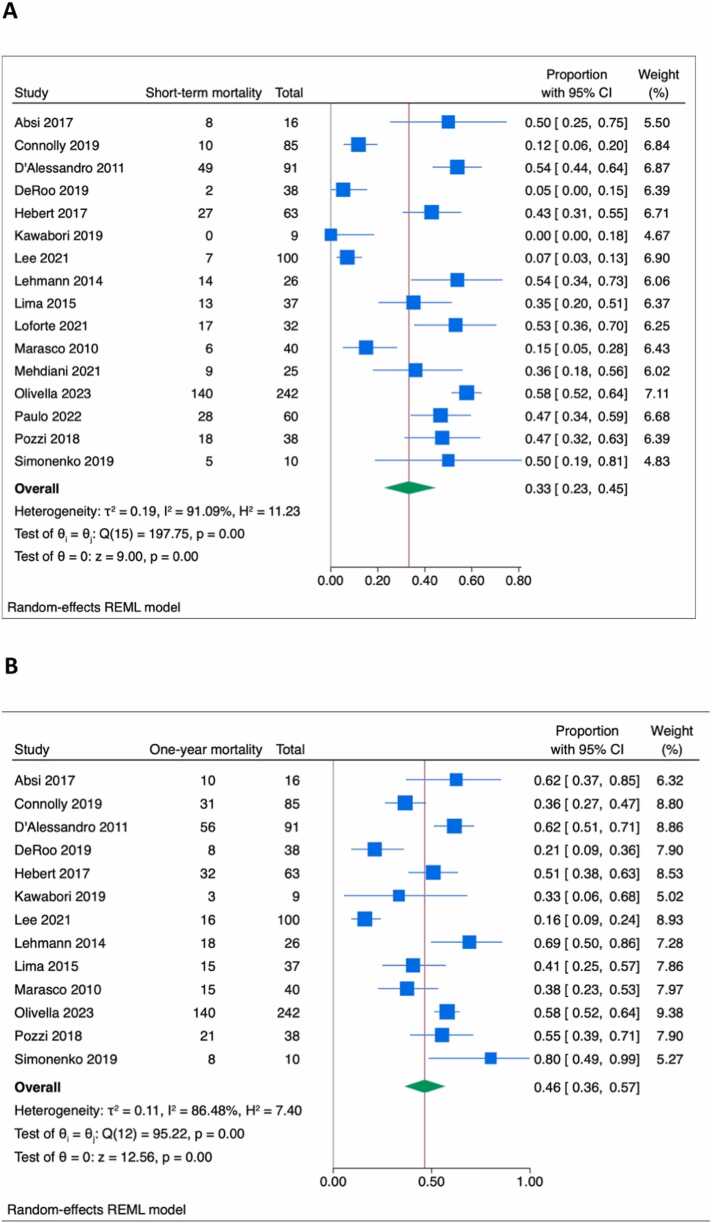
Forest plot of short-term (A) and 1-year (B) mortality. CI, confidence interval.

**Figure 2:**
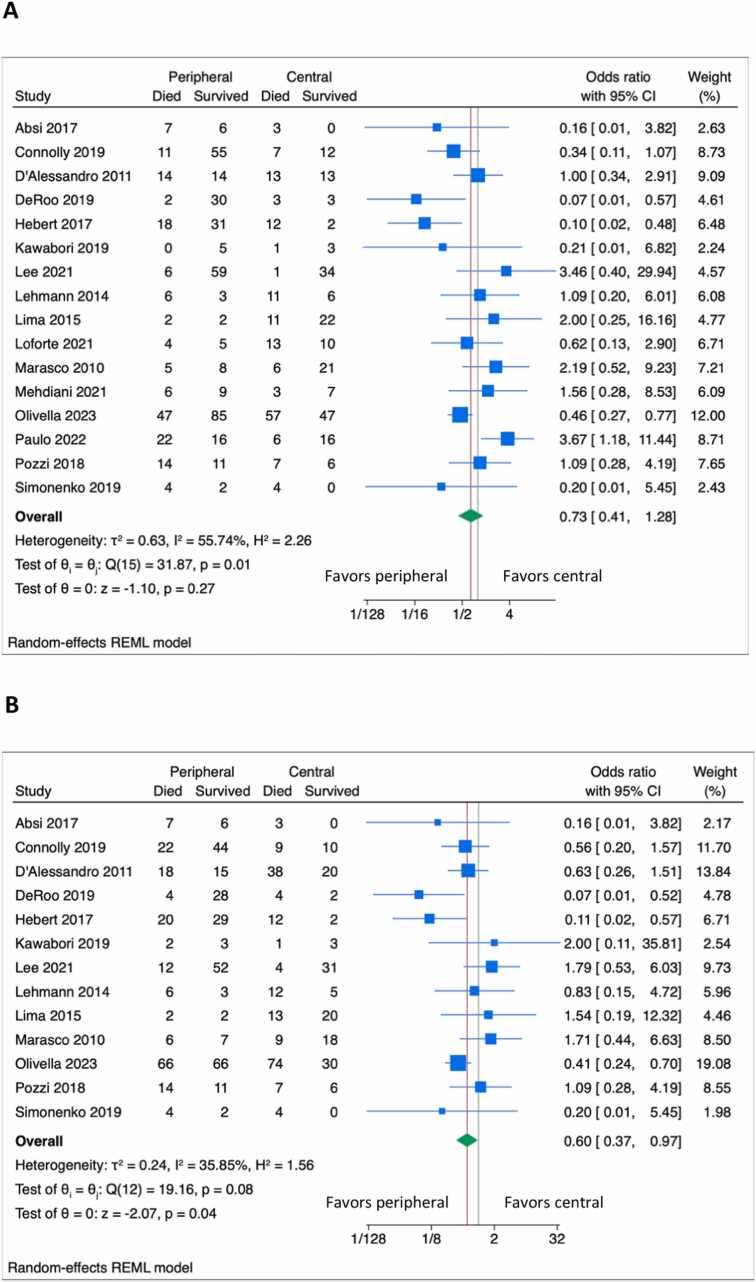
Forest plot of short-term (A) and 1-year (B) mortality according to the use of peripheral vs central VA-ECMO cannulation. Right favors central cannulation; left favors peripheral cannulation. CI, confidence interval; VA-ECMO, veno-arterial extracorporeal membrane oxygenation.

The pooled estimate for short-term mortality was 33% (95% confidence interval [CI] = 23%-45%; I2 = 91.1%) from 16 studies (Figure 1). Multicentered studies reported higher short-term mortality than single-center studies (51% vs 31%, *p* = 0.03). Heterogeneity was not explained by using the ISHLT definition for PGD nor by using IPD data (*p* = 0.47 and *p* = 0.29 for the respective interactions). Peripheral cannulation was associated with a nonsignificant reduction in short-term mortality (OR = 0.73, 95%CI = 0.41-1.28, I2 = 55.8%, Figure 2). Duration of VA-ECMO support, reported in 11 studies, was similar between groups (Cohen’s *d* standardized mean difference of −0.14 days in peripheral cannulation, 95%CI −0.39 to 0.11 days, I2 = 33.0%). The length of stay was reported in 6 studies and was similar (0.15 days longer in peripherally cannulated patients, 95%CI = −0.30 to 0.61, I2 = 50.5%).

The pooled estimate for 1-year mortality was 46% (95%CI = 36%-57%) from 13 (81.3%) studies. Heterogeneity was not explained by single vs multicenter studies (*p* = 0.10), by using the ISHLT definition for PGD (*p* = 0.37) nor by using IPD data (*p* = 0.86). Peripheral cannulation was associated with reduced 1-year mortality compared to central cannulation (OR = 0.60, 95%CI = 0.37-0.97, I2 = 35.9%).

The certainty of evidence supporting peripheral over central cannulation was low given the observational nature of the studies, imprecision, and moderate heterogeneity.

**Figure 3:**
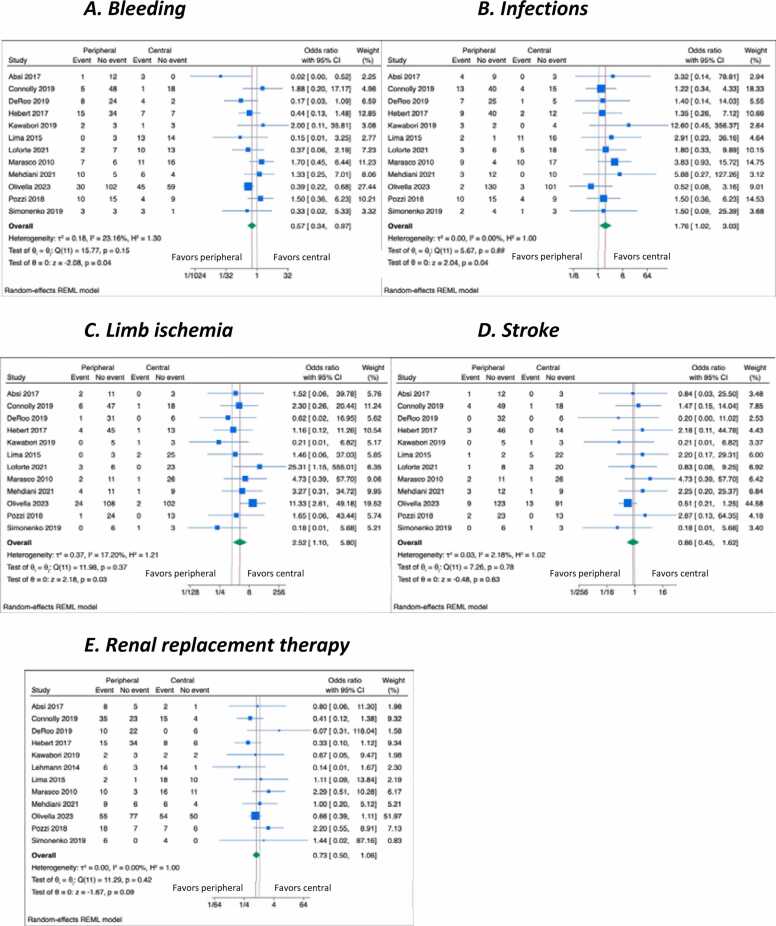
Forest plot of bleeding (A), infections (B), limb ischemia (C), stroke (D), or RRT (E) mortality according to the use of peripheral vs central VA-ECMO cannulation. RRT, renal replacement therapy; VA-ECMO, veno-arterial extracorporeal membrane oxygenation.

VA-ECMO–related complications were reported in 12 studies (75%, Figure 3). The pooled estimate for bleeding was 37% (95%CI = 27%-46%, I2 = 78.9%), and was lower in patients with peripheral cannulation (OR = 0.57, 95%CI = 0.34-0.97, I2 = 23.2%). Infections occurred in 30% (95%CI = 23%-38%, I2 = 68.2%), more frequently among patients supported with peripheral cannulation (OR = 1.76, 95%CI = 1.02-3.03, I2 = 0%). The pooled estimate for limb ischemia was 8% (95% CI 6%-11%, I2 = 0.0%) and was significantly higher with peripheral cannulation (OR = 2.52, 95%CI = 1.10-5.80, I2 = 17.2%). RRT was required in 60% of cases (95%CI = 48%-71%, I2 = 86.0%), with no difference between cannulation strategies (OR = 0.73, 95%CI = 0.50-1.06, I2 = 0.0%). Stroke was observed in 7% (95%CI = 4%-10%, I2 = 31.1%), with no significant differences (OR = 0.86, 95%CI = 0.45-1.62, I2 = 2.2%).

## Discussion

In this meta-analysis of 874 patients requiring VA-ECMO for PGD, peripheral cannulation may reduce 1-year mortality, although less certainty exists regarding short-term mortality. Peripheral cannulation was associated with less bleeding, similar rates of stroke and RRT, but more infections and limb ischemia. The certainty of these estimates is low given the risk of bias, imprecision, and heterogeneity (Table S2).

Our work expands to the HT population results from previous works including patients requiring VA-ECMO in non-HT postcardiotomy shock, which demonstrated lower mortality rates and lower risk of bleeding with peripheral configuration.^2^ Central VA-ECMO is still used in 30% to 50% of cases in postcardiotomy shock^2^, ^9^; it uses cannulas in place for cardiopulmonary bypass and avoids harlequin syndrome, but it defers extubation, requires chest re-entry and increases bleeding and mediastinitis. Small case series in post-HT setting have yielded conflicting results and a meta-analysis identified a trend toward improved survival with cannulation.^4^ However, a large, multicentric study of 242 demonstrated a reduction in mortality with peripheral cannulation after adjustment for donor and recipient variables, ischemic time, and transplant era.^5^

As limitations, we did not include studies with postcardiotomy shock that did not report separately patients with PGD nor articles that did not report data regarding cannulation site. The evidence available is exclusively observational, mostly retrospective with no randomized data, and the decision to cannulate peripherally or centrally may be driven by patient factors and center protocols. Rates of mediastinitis, which one would expect could negatively impact outcomes in patients with central cannulation, were lacking in most studies, and could therefore not be compared between groups. The timing (early vs deferred) and place of cannulation (operating room vs intensive care unit), which might have also influenced the observed outcomes, could not be assessed as a potential factor mediating the observed results. Also, none of the studies reported on left ventricular venting strategies during VA-ECMO support, and its impact on prognosis after PGD remains unexplored.

## Conclusions

With low certainty evidence, peripheral VA-ECMO cannulation may reduce short-term and 1-year mortality, is associated with lower bleeding, similar rates of stroke and RRT, and more frequent limb-related complications and infections. These results support the use of a peripheral configuration in HT recipients with PGD.

## Author contributions

Conceptualization: E.R.-A., A.O., N.A. Methodology: E.R.-A., N.A. Formal analysis: E.R.-A. Investigation: E.R.-A., A.O., A.O.-C., N.A. Resources: H.J.R., A.C.A., N.A. Writing—original draft: E.R.A., A.O. Writing—review and editing: A.O.-C., F.F., Y.M., V.R., F.B., H.J.R., A.C.A., N.A. Visualization: E.R.-A., A.O., N.A. Supervision: N.A. Project administration: N.A. Funding acquisition: N.A., H.J.R.

## Disclosure statement

The authors declare that they have no known competing financial interests or personal relationships that could have appeared to influence the work reported in this paper.

Eduard Ródenas-Alesina has received travel support from Abbott. Filio Billia has received grant support from Abbott for a physician-initiated study. Vivek Rao has received honoraria from Abbott and Medtronic, fees as a consultant for Medtronic, has been in the surgical advisory board for Medtronic, and has invested in Medtronic Equity stock.

No funding was received for this study.

## Footnotes

Appendix A Supplementary data associated with this article can be found in the online version at doi:10.1016/j.jhlto.2024.100174.
