# Supplementary material for: Peripheral versus central cannulation of VA-ECMO for primary graft dysfunction after heart transplantation: A systematic review and meta-analysis
Source: JHLT Open. 2024 Nov 8;7:100174. doi: 10.1016/j.jhlto.2024.100174 (PMC11935498; doi:10.1016/j.jhlto.2024.100174)

**Supplementary Table S1: Risk of bias assessment using the ROBINS-I tool for the effect of VA-ECMO cannulation strategy**

| Study | Outcome | Overall risk of bias | Confounding | Selection of participants | Classification of intervention | Deviation from intended intervention | Missing data | Measurement of outcome | Selection of the reported results |
| --- | --- | --- | --- | --- | --- | --- | --- | --- | --- |
| Marasco 2010 | Death by hospital discharge |  |  |  |  |  |  |  |  |
| D’Alessandro 2011 | Death by hospital discharge |  |  |  |  |  |  |  |  |
| Lehmann 2014 | Death by hospital discharge |  |  |  |  |  |  |  |  |
| Lima 2015 | Death by hospital discharge |  |  |  |  |  |  |  |  |
| Absi 2017 | Death by hospital discharge |  |  |  |  |  |  |  |  |
| Hebert 2017 | Death by hospital discharge |  |  |  |  |  |  |  |  |
| Pozzi 2018 | Death by hospital discharge |  |  |  |  |  |  |  |  |
| Kawabori 2019 | Death by hospital discharge |  |  |  |  |  |  |  |  |
| Simonenko 2019 | Death by hospital discharge |  |  |  |  |  |  |  |  |
| DeRoo 2019 | Death by hospital discharge |  |  |  |  |  |  |  |  |
| Connolly 2019 | Death by hospital discharge |  |  |  |  |  |  |  |  |
| Mehdiani 2021 | 30-day mortality |  |  |  |  |  |  |  |  |
| Lee 2021 | 30-day mortality |  |  |  |  |  |  |  |  |
| Loforte 2021 | Death by hospital discharge |  |  |  |  |  |  |  |  |
| Paulo 2022 | Death by hospital discharge |  |  |  |  |  |  |  |  |
| Olivella 2023 | 30-day mortality |  |  |  |  |  |  |  |  |

| No information | Low | Moderate | Serious | Critical |
| --- | --- | --- | --- | --- |

**Supplementary Table S2.** **Summary of findings for prognosis: mortality and VA-ECMO complications based on cannulation strategies**

| **№ of studies** | **Certainty assessment** | | | | | **Certainty** | **Importance** |
| --- | --- | --- | --- | --- | --- | --- | --- |
|  | **Risk of bias** | **Inconsistency** | **Indirectness** | **Imprecision** | **Other considerations** |  |  |
| **Short-term mortality (in hospital or 30 days mortality if not reported)** | | | | | | | |
| 16 | serious^a^ | serious | not serious | not serious | none^e^ | ⨁⨁◯◯  Low | CRITICAL |
| **1-year mortality** | | | | | | | |
| 13 | serious^a^ | serious | not serious | not serious | none^e^ | ⨁⨁◯◯  Low | CRITICAL |
| **VA-ECMO complication: bleeding** | | | | | | | |
| 12 | serious^b^ | not serious^d^ | not serious | not serious | none | ⨁⨁◯◯  Low | IMPORTANT |
| **VA-ECMO complication: infection** | | | | | | | |
| 12 | serious^b^ | not serious^d^ | not serious | not serious | none | ⨁⨁◯◯  Low | IMPORTANT |
| **VA-ECMO complication: stroke** | | | | | | | |
| 12 | serious^b^ | not serious^d^ | not serious | not serious | none | ⨁⨁◯◯  Low | IMPORTANT |
| **VA-ECMO complication: limb ischemia** | | | | | | | |
| 12 | serious^b^ | not serious^d^ | not serious | not serious | none | ⨁⨁◯◯  Low | IMPORTANT |
| **Need for dialysis** | | | | | | | |
| 12 | serious^a^ | serious^a^ | not serious | not serious | none | ⨁⨁◯◯  Low | IMPORTANT |

#### Explanations

a. Study confounding, b. Substantial unexplained heterogeneity, c. Outcome not well defined, study confounding, d. Heterogeneity can be explained by differences in outcome definitions, which is accounted for in the risk of bias assessment, e. Publication bias judged not significant because of the unreliability of Egger’s test for observational data of proportions and overall symmetrical appearance of the funnel plots

**Supplementary Figure S1**

**Figure S1-A.** Funnel plot for short-term mortality **Figure S1-B.** Funnel plot for one-year mortality


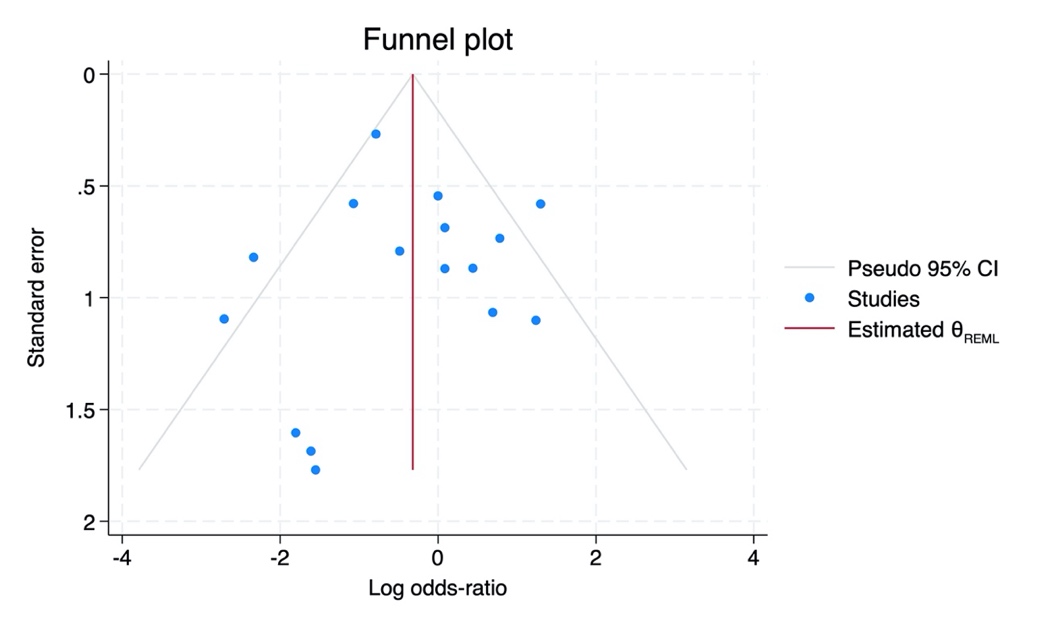

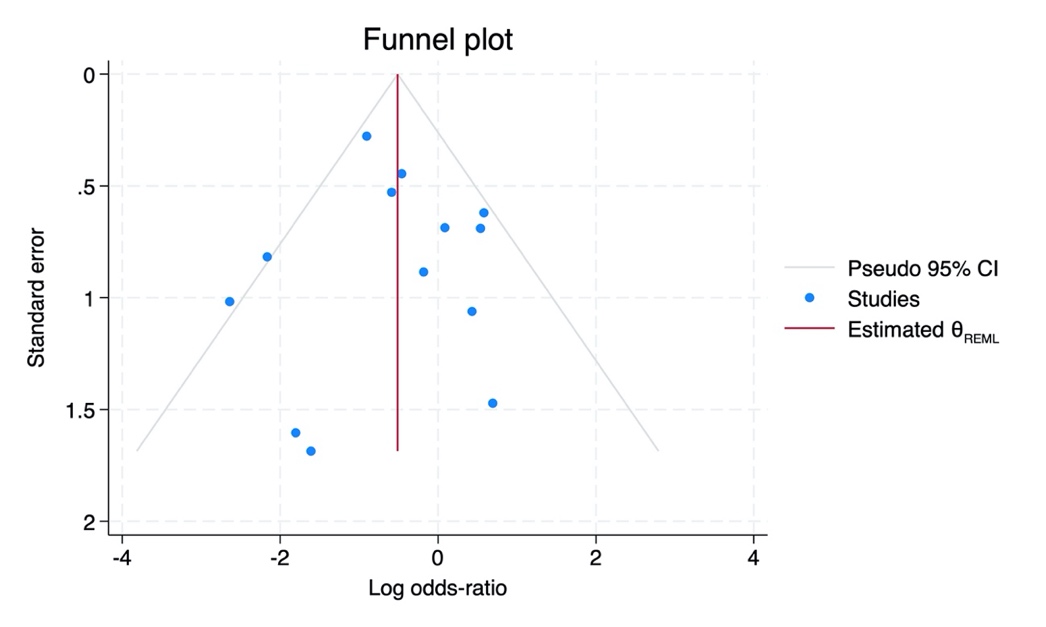

Supplement: Supplementary file 1 — Supplementary material [file mmc1.docx]
